# Assessing the Aflatoxin B_1_ Adsorption Capacity between Biosorbents Using an In Vitro Multicompartmental Model Simulating the Dynamic Conditions in the Gastrointestinal Tract of Poultry

**DOI:** 10.3390/toxins10110484

**Published:** 2018-11-21

**Authors:** Anai Zavala-Franco, Daniel Hernández-Patlán, Bruno Solís-Cruz, Raquel López-Arellano, Guillermo Tellez-Isaias, Alma Vázquez-Durán, Abraham Méndez-Albores

**Affiliations:** 1 Center for Research and Advanced Studies of the National Polytechnic Institute (CINVESTAV-IPN), Real de Juriquilla, Queretaro 76230, Mexico; anai.zavala@cinvestav.mx; 2 National Autonomous University of Mexico-Superior Studies Faculty at Cuautitlan (UNAM–FESC), Multidisciplinary Research Unit L5, Cuautitlan Izcalli 54714, Mexico; danielpatlan@comunidad.unam.mx (D.H.-P.); bruno_sc@comunidad.unam.mx (B.S.-C.); lopezar@unam.mx (R.L.-A.); 3 Department of Poultry Science, University of Arkansas, Fayetteville, AR 72701, USA; gtellez@uark.edu; 4 National Autonomous University of Mexico-Superior Studies Faculty at Cuautitlan (UNAM–FESC), Multidisciplinary Research Unit L14 (Food, Mycotoxins, and Mycotoxicosis), Cuautitlan Izcalli 54714, Mexico; almavazquez@comunidad.unam.mx

**Keywords:** aflatoxin B_1_, decontamination, biosorbents, in vitro digestion model

## Abstract

Experiments were carried out to evaluate the effectiveness of three different biosorbents (banana peel, *Pyracantha* leaves, and *Aloe* powder) in removing aflatoxin B_1_ (AFB_1_). A noncommercial mycotoxin binder (zeolite) was used as a reference material. A laboratory model that simulated the in vivo conditions of the poultry gastrointestinal tract was utilized to prove the removal efficiency of the biosorbents when added to AFB_1_-contaminated diet (100 µg/kg). The concentration of AFB_1_ was determined using antibody-based immunoaffinity column and spectrofluorometry methodologies. Z potential (ζ), point of zero charge (pH_pzc_), energy-dispersive X-ray spectroscopy (EDS), X-ray diffraction (XRD), Fourier transform infrared spectroscopy with attenuated total reflection (FTIR-ATR), and UV-Vis diffuse reflectance spectroscopy (DRS) techniques were used to further characterize the biosorbents. The addition of the biosorbents (1.5%, *w*/*w*) to the diet significantly reduced the bioavailability of AFB_1_ in the intestinal section. The highest aflatoxin adsorption values were 69% and 70% using *Aloe* powder and zeolite, respectively. A moderate biosorption uptake of 46% was achieved using *Pyracantha* leaves. The biomaterial with the lowest removal capacity was banana peel (28%). In conclusion, *Aloe* powder could be used as an alternative to conventional systems for AFB_1_ removal.

## 1. Introduction

The Food and Agriculture Organization of the United Nations (FAO) estimated that 25% of the cereal production is contaminated by mycotoxins [1]. Aflatoxin B_1_ (AFB_1_)—the major secondary metabolite of *Aspergillus flavus*, *A. parasiticus*, and *A. nomius*—has adverse effects on humans and animals that result in health disorders and economic losses. To avoid these harmful effects, many physical, chemical, and biological decontamination strategies have been proposed. The most used technique is the inclusion of adsorbent materials into contaminated diets in order to be useful in the gastrointestinal (GI) tract. In the poultry industry, inorganic compounds such as aluminosilicates or zeolites, hydrated sodium calcium aluminosilicate (HSCAS), and aluminosilicate-containing clays are the most commonly used adsorbent materials [2]. However, there are some risks associated with them, such as their ability to indiscriminately adsorb some vitamins and minerals or the possibility that they release toxic components, such as heavy metals or dioxins [3].

Recently, biosorption technology has appeared as a favorable alternative over conventional adsorbent materials, with the potential benefits of low cost, greater profitability, practicality, and good efficiency. It has been reported that biomasses obtained from *Pyracantha coccinea* berries, banana peels, and orange peels are capable of removing different compounds such as dyes and phenolic compounds [4,5,6]. Our research group reported that biomasses derived from *Pyracantha koidzumii* (leaves and berries) were able to remove B-aflatoxins in aqueous solutions [7]. Furthermore, it has been described that banana peel may be an effective and low-cost biosorbent against AFB_1_ removal [8]. Different in vitro methodologies have been reported to evaluate the aflatoxin-adsorbent capacity of the organic binders. However, these methodologies may not be directly applicable to poultry due to the fact that they do not use consecutive incubation at different pH values, temperature, and enzymatic activities under in vivo circumstances. Although *Aloe vera* has been described to have several properties, such as virucidal, bactericidal, and fungicidal as well as the adsorbent characteristic against heavy metals in aqueous solution [9], there are no reports of its use as a biosorbent against AFB_1_. Therefore, the present study was conducted to characterize and evaluate the biosorption potential of banana peel, *Pyracantha* leaves, and *Aloe* powder in order to propose an unconventional, eco-friendly, and efficient binder for AFB_1_ adsorption using a multicompartmental model simulating the kinetic conditions in the GI tract of poultry.

## 2. Results and Discussion

### 2.1. Zeta Potential and Point of Zero Charge

**Figure 1:**
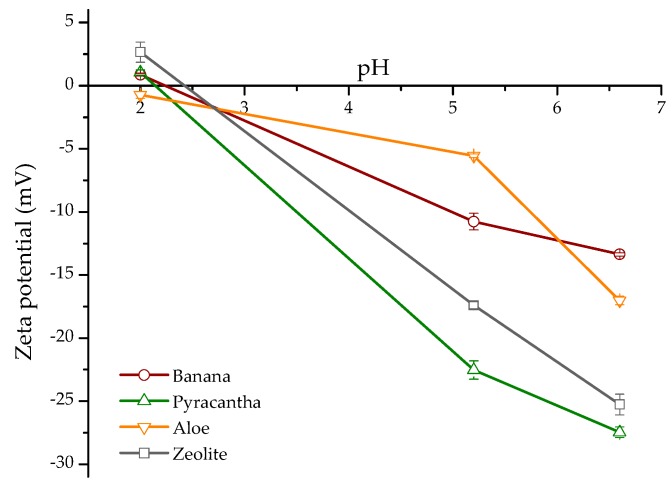
The relationship between zeta potential (ζ) and pH of biosorbents and the inorganic mycotoxin binder (zeolite). Mean values ± standard error.

Zeta potential is the electrokinetic potential in colloidal systems and can be used to characterize the surface of charged particles [10]. Both surface charge and environmental conditions can influence the zeta potential. As a result, pH and ions in the medium may alter the zeta potential [11]. In this research, a zeta potential value of −5.9 mV was observed in *Aloe* powder. However, for banana peel and *Pyracantha* leaves, the zeta potential values were −12.7 mV and −20.9 mV, respectively. The zeolite presented an average zeta potential value of −16.7 mV. These results are in accordance with Ma et al. [12] and Ramales-Valderrama et al. [7] who reported zeta potential values of −16.2 mV and −21.8 mV for banana peel and *Pyracantha* leaves, respectively. Figure 1 shows the zeta potential of the biosorbents and the zeolite at three different pH values of the in vitro digestive model. The first GI compartment simulated was the crop (pH~5.2), the next compartment was the proventriculus (pH~1.7), and the third was the intestinal section (pH~6.7). In general, zeta potential increased significantly with increasing pH value and reached the maximum at pH 6.7. Under these circumstances, the high negative-charged surface on the biosorbents could result in a high sorption uptake in the in vitro digestion model due to possible improvements of attractive forces between AFB_1_ and the biosorbent surface. Consequently, the interaction type between AFB_1_ and the biosorbents in some GI compartments, such as the intestinal section, would be mainly electrostatic considering that AFB_1_ is a very polar molecule with a substantially high positive charge [5]. Interestingly, *Aloe* powder exhibited a negative zeta potential value at the three different pH values tested.

**Figure 2:**
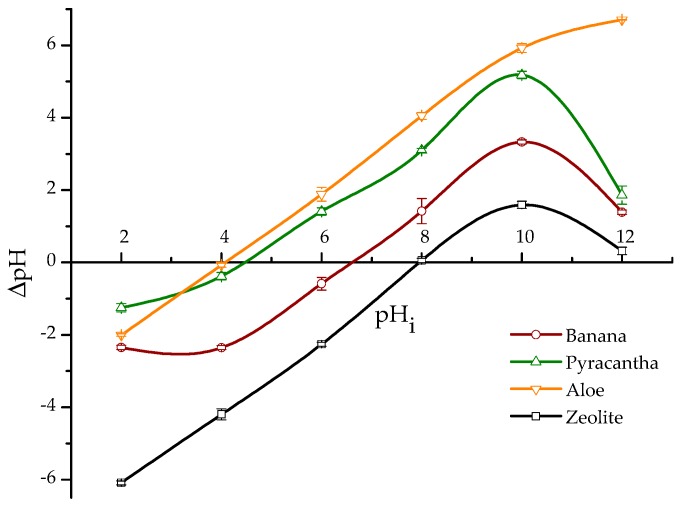
Point of zero charge (pH_pzc_) of biosorbents and the inorganic mycotoxin binder (zeolite). Mean values ± standard error.

Point of zero charge (pH_pzc_) provides information about the surface charge of the biosorbents. The pH_pzc_ was calculated by plotting ΔpH against the initial pH value (Figure 2). In the case of *Aloe* powder and *Pyracantha* leaves, the curves cross the horizontal line at pH values of 4.1 and 4.5, respectively. However, for banana peel and the zeolite, the curves cross the horizontal line at pH 6.7 and 7.9, respectively. Therefore, the surface charge of these sorbents is zero at those pH values. It is well known that if pH < pH_pzc_, the surface will be positively charged, and if pH > pH_pzc_, the surface is negatively charged [13]. In this regard, the pH_pzc_ of banana peel was reported to be 5.5 by Shar et al. [8]. Bhaumik and Mondal [14] reported pH_pzc_ values from 6.2 to 8.2 for banana peel sun-dried for 12 h followed by drying at 50 °C for 24 h. These slight variations in pH_pzc_ might be due to the differences in banana species/cultivar or stage of ripeness. It is quite important to note that *Aloe* powder and *Pyracantha* leaves have a high negative-charged surface at the pH value of the intestinal section; consequently, it is expected that these materials have significant biosorption uptakes in the in vitro digestive model. Considering the pH of the final GI compartment and the positive charge of the aflatoxin molecules, biosorbents with low values in the pH_pzc_ aspect are the most appropriate to be used for the adsorption process.

It is well known that zeolites do not exhibit a constant negative charge in the pH range from 4 to 7, which is attributed to a permanent charge arising from the isomorphous substitutions in the crystal structure [15]. In this research, the presence of only pH-dependent positive charge on the zeolite is consistent with the results obtained by Houng et al. [16]. This positive charge arises from the protonation of the hydrous oxide surface on the acid side of the pH_pzc_. Consequently, no negative charges would be expected when the pH of the zeolite suspension is less than the pH_pzc_, which is 7.9 (Figure 2). However, aflatoxin adsorption into inorganic adsorbents is also mediated by other mechanisms, such as weakly electrostatic attractions, moderate electron donor–acceptor attraction, and a robust calcium-bridging linkage [17].

### 2.2. Multielemental Analysis

**Figure 3:**
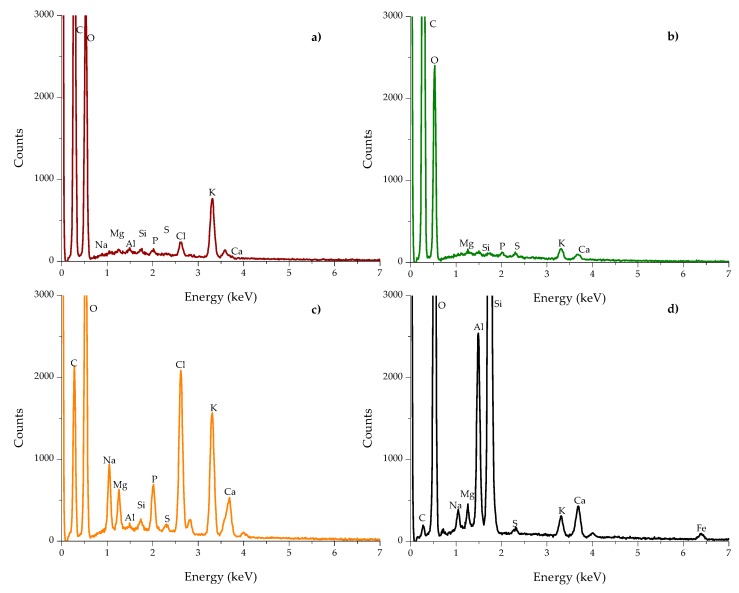
Energy-dispersive X-ray spectroscopy (EDS) spectra of (**a**) banana peel, (**b**) *Pyracantha* leaves, (**c**) *Aloe* powder, and (**d**) the inorganic mycotoxin binder (zeolite).

Figure 3 shows the energy-dispersive X-ray spectroscopy (EDS) spectra of the biosorbents and the zeolite. The percentage of the elements is presented in Table 1. The main elements of the three organic sorbents were C and O, accounting for 97.3%, 99.2%, and 85.7% of the total weight of the banana peel, *Pyracantha* leaves, and *Aloe* powder, respectively. Other minor elements were involved in *Aloe* powder, such as Na (2.08%), P (1.09%), Cl (4.27%), K (4.10%), and Ca (1.47%). In general, no statistical differences regarding Al and Si composition were observed for banana peel and *Aloe* powder; however, in the case of Na, Mg, P, S, Cl, K, and Ca, there were statistically significant differences (Table 1). Fe was not detected in biosorbents. *Aloe* powder and the zeolite presented similar elemental composition regarding S and Ca. The chemical composition of certain biomasses has been previously reported. Memon et al. [18] performed elemental analysis of banana peel and found that the element with high content was K, while low contents of Si, Ca, Na, Al, and Mg were also observed. Similarly, Achak et al. [4] reported higher contents of K in the chemical composition of banana peel. Khaniabadi et al. [19] reported that the principal elements in sulfuric-acid-modified activated carbon adsorbent in wastes of *Aloe vera* leaves were O, Ca, K, Mg, Na, and Cl. These results are consistent with those found in this research. As can be seen, the zeolite was possessed with high contents of oxygen (65.10%), silicon (20.86%), aluminum (5.29%), calcium (1.45%), sodium (0.97%), potassium (0.75%), and iron (0.59%), and low contents of sulfur (0.19%). Phosphorous and chlorine were not detected. It is well known that zeolites are regularly composed of oxygen, silicon, and aluminum [20]. Other authors have reported similar chemical composition for this kind of material [21,22].

### 2.3. X-ray Diffraction (XRD)

**Figure 4:**
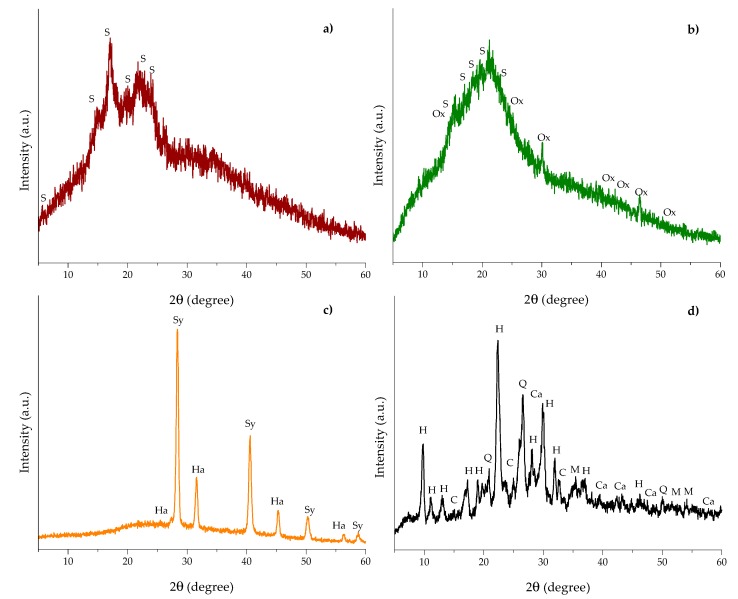
Representative X-ray diffraction patterns of (**a**) banana peel, (**b**) *Pyracantha* leaves, (**c**) *Aloe* powder, and (**d**) the inorganic mycotoxin binder (zeolite). S = starch, Ox = calcium oxalate, Ha = halite, Sy = sylvite, H = heulandite, C = clinoptilolite, Q = quartz, Ca = calcite, M = magnetite.

XRD has the advantage of a good fingerprint nondestructive characterization and is also a suitable technique to study phase and crystal structure. Figure 4 shows the XRD patterns of the biosorbents and the zeolite. In general, banana peel and *Pyracantha* leaves presented the characteristic amorphous structure (Figure 4a,b), while *Aloe* powder and the zeolite showed both the amorphous and crystalline phases (Figure 4c,d).

Banana peel and *Pyracantha* leaves showed the strongest diffraction peak at 20.0° 2θ and few small diffraction peaks at around 5.6, 14.8, 17.3, 22.8, and 24.0°. These diffraction peaks are related to the structure of semicrystalline starch. Banana peel had diffraction peaks similar to those of potato starch [(C_6_H_10_O_5_)_n_, standard card JCPDS 052-2247], and *Pyracantha* leaves gave the same peaks as that of wheat starch [(C_6_H_10_O_5_)_n_, standard card JCPDS 053-1663]. In addition, *Pyracantha* leaves showed diffraction peaks associated with calcium oxalate [(COO)_2_ Ca·H_2_O, standard card JCPDS 014-0768]. Starch is a semicrystalline polymer with crystallinity between 15% and 45% [23]. In this research, the degree of crystallinity was calculated according to the recommendations of Shujun et al. [24]. The degree of crystallinity of the banana peel and *Pyracantha* leaves differed significantly; *Pyracantha* leaves showed the lowest degree of crystallinity (10.9%), whereas banana peel exhibited a moderate degree of crystallinity (19.1%). In general, starch crystallinity depends on the size of the crystal, number of crystalline regions, and orientation of the crystalline domains. Few studies have presented the peaks diffracted by banana peel [25,26,27,28], and the results are in accordance with those reported in this research. Moreover, the corresponding X-ray diffraction parameters for *Aloe* powder showed an amorphous region and also crystalline peaks for sylvite (KCl, JCPDS: 004-0587) at 27.3, 31.6, 45.3, and 56.4° 2θ, and halite (NaCl, JCPDS: 005-0628) at 28.3, 40.6, 50.3, 58.8, and 66.7° 2θ. Apparently, due to the presence of trace amounts of these salts, *Aloe* powder shows a high degree of crystallinity (44.7%). Furthermore, the zeolite presented several diffraction peaks corresponding to different crystalline structures, such as heulandite (Ca_3.6_K_0.8_Al_8.8_Si_27.4_O_72_·26.1H_2_O, standard card JCPDS 053-1176), clinoptilolite (Na_5_Al_6_Si_30_O_72_·18H_2_O, standard card JCPDS 047-1870), quartz (SiO_2_, standard card JCPDS 01-089-1961), calcite (CaCO_3_, standard card JCPDS 047-1743), and magnetite (Fe_3_O_4_, standard card JCPDS 019-0629). Zeolites are hydrated aluminosilicate compounds formed by large intersecting open channels of the tetrahedral rings. The lattice consists of tetrahedra of silicate (SiO_4_) and aluminate (AlO_4_) joined together by oxygen atoms. Because aluminum is trivalent, the lattice is charged negatively; however, this charge is neutralized by positively charged ions such as Na, K, Ca, or Fe. It is well known that clinoptilolite is a structural variant of heulandite, which is differentiated by the Si:Al ratio. Those with Si:Al ratio <4 are known as heulandite, and those with Si:Al ratio >4 are recognized as clinoptilolite [29]. In this research, the zeolite was mainly heulandites because the Si:Al ratio was 3.94 (Table 1). This inorganic mycotoxin binder had a higher degree of crystallinity (50.2%) than the organic binders, which is in accordance with results reported previously by Behin et al. [30].

### 2.4. Infrared Spectroscopy

**Figure 5:**
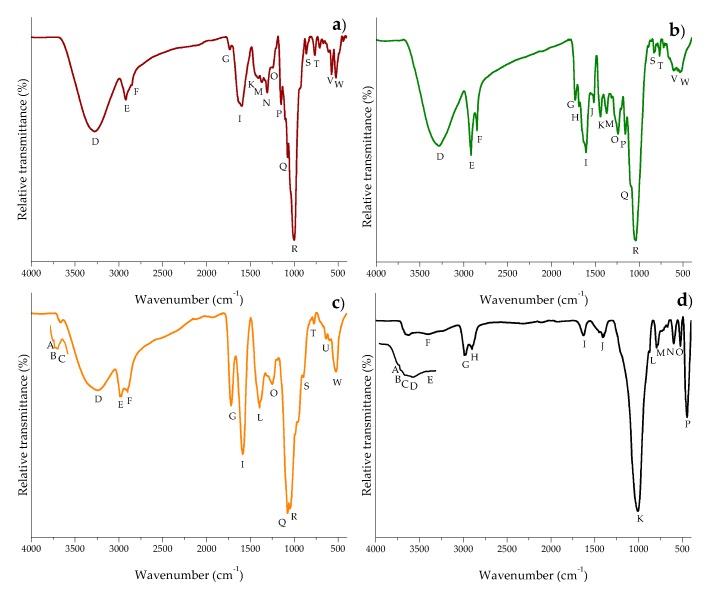
Comparative Fourier transform infrared spectra of (**a**) banana peel, (**b**) *Pyracantha* leaves, (**c**) *Aloe* powder, and (**d**) the inorganic mycotoxin binder (zeolite).

To identify the specific functional groups, Fourier transform infrared spectroscopy with attenuated total reflection (FTIR-ATR) studies were conducted in the three different biosorbents and the zeolite. A FTIR spectra comparison is showed in Figure 5. Table 2 and Table 3 indicate the main FTIR bands and their corresponding assignations. It can be noticed that higher percentages of transmittance meant lower quantities of functional groups.

In general, significant differences in the primary active vibrations were noted among the biosorbents. *Aloe* powder and the zeolite showed the characteristic bands at around 3685, 3674, and 3660 cm^−1^, respectively, associated with the stretching vibrations of water molecules bound to metals such as Al and Mg [31]. Furthermore, the three biosorbents exhibited higher quantities of functional groups associated with the hydroxyl, carboxyl, amide, phosphate, and ketone groups (Figure 4a–c). These groups played the significant role of adsorption in the elimination of aflatoxin, which is in agreement with our previous work [7]. The zeolite also showed two distinctive bands at 3624 and 3564 cm^−1^ due to the presence of Al^3+^–OH in the octahedral sheet and to the octahedral Fe^3+^–OH, respectively [31,32]. There was also a strong evidence that the zeolite was hydrated, illustrated by discrete water absorption bands at 3568 and 1631 cm^−1^, respectively. These bands refer to water molecules associated with Na and Ca in the channels and cages of the clay structure. The other band at 1013 cm^−1^ is related to the stretching vibration of T–O in TO_4_ tetrahedra (T = Si and Al). The 794 and 465 cm^−1^ bands are attributed to the stretching vibration of O–T–O and the bending of T–O bonds, respectively [33]. Finally, the vibration at 597 cm^−1^ is generally associated with the presence of heulandite [34].

### 2.5. Diffuse Reflection UV-Vis Spectroscopy

**Figure 6:**
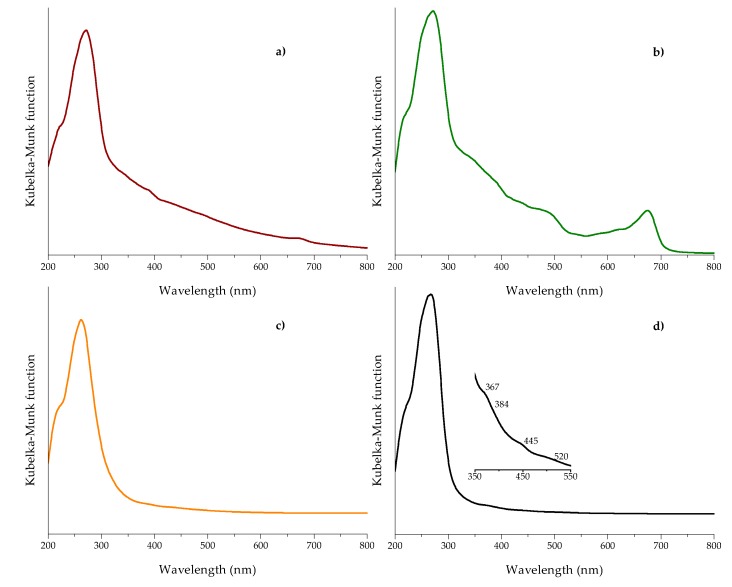
UV-Vis diffuse reflectance spectra after Kubelka–Munk treatment of (**a**) banana peel, (**b**) *Pyracantha* leaves, (**c**) *Aloe* powder, and (**d**) the inorganic mycotoxin binder (zeolite).

The UV-Vis diffuse reflectance spectroscopy (DRS) is a convenient technique to characterize different materials, including plant tissues. The detection of absorbance bands in these materials is governed predominantly by the pigment content, such as chlorophylls, carotenoids, and anthocyanins. Figure 6 shows the DR spectra of the different biosorbents and the zeolite after Kubelka–Munk treatment. In general, *Pyracantha* leaves and banana peel presented the characteristic absorption band in the red region (677 nm), which corresponds to chlorophyll *a* [35]. *Pyracantha* leaves also showed a shoulder near 650 nm, related to the presence of chlorophyll *b*. Other compounds that could explain the increment of absorbance in *Pyracantha* leaves are carotenoids and anthocyanins. Carotenoids have an absorbance band in the blue region (425 to 485 nm), and anthocyanins at 350 to 730 nm, with a shoulder around 550 nm (Figure 6b). *Aloe* powder did not present any particular band.

The UV-Vis DRS is also a useful technique to elucidate some structural, physical, and chemical properties of clay materials. In general, the basic structural SiO_4_ units of the zeolite do not absorb light in the range of 200 to 800 nm; absorption peaks in this region are due to the presence of transition metals. It is well known that clay materials contain large amounts of transition metals, but the electronic transitions in the ultraviolet and visible regions are almost without exception associated with the presence of iron [36]. In this research, the zeolite showed the principal absorption band centered at approximately 262 nm (Figure 6d). This very strong band is commonly assigned to Fe^3+^ ← O^2−^, OH^−^ or OH_2_ charge transfer [36,37]. Furthermore, the zeolite showed not only the ultraviolet charge transfer band related to the octahedral Fe^3+^, but also showed intraconfigurational transitions in the visible region. These minor contributions were a moderate shoulder at 367 nm, a weak shoulder at 384 nm, a strong shoulder at 445 nm, and a broad shoulder at 520 nm (inset of Figure 6d)—all of them are associated with the presence of iron in the clay material [36].

### 2.6. Adsorption Experiments

Results of the biosorption of AFB_1_ are shown in Table 4. The control group—not added with adsorbents—had an aflatoxin value of 27 ng/mL, denoting the lack of AFB_1_ adsorption. Conversely, *Aloe* powder showed the highest efficiency of the biosorbents against aflatoxin removal, the biosorption uptake was 68.52%, statistically similar to those observed in the zeolite (70.19%). In these samples, the final aflatoxin contents were 8.5 and 8.0 ng/mL, respectively. In contrast, *Pyracantha* leaves showed a modest aflatoxin uptake capacity, reaching values up to 46.30%. The biomaterial with the lowest adsorbent capacity was banana peel (27.78%).

**Figure 7:**
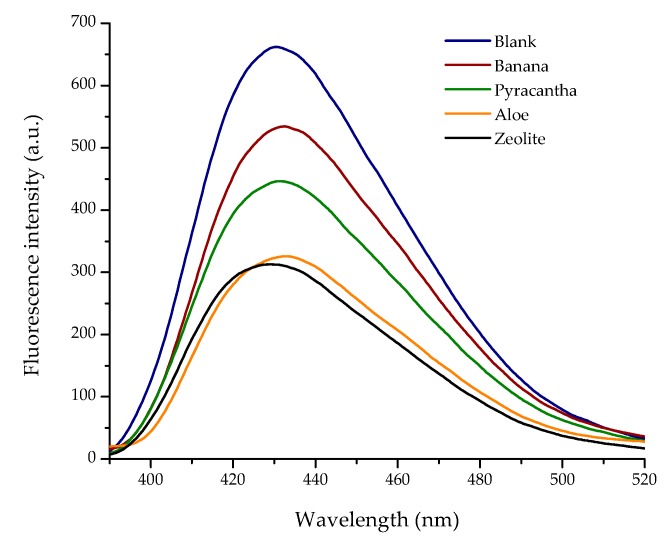
Influence of the different biosorbents and the inorganic mycotoxin binder (zeolite) on the fluorescence spectra of aflatoxin B_1_ (AFB_1_).

In general, using the complete in vitro digestion model, the sorption capacity of the biomaterials varied considerably as seen in their corresponding fluorescence spectrum (Figure 7). Various biosorbents, such as *Pyracantha koidzumii* [7], banana peel (Cavendish) [8], yeast and yeast products [38], agricultural byproducts [39], and grape pomace (pulp and skins) [40] have been tested for mycotoxin removal using different in vitro methodologies. However, these methodologies may not be directly applicable to poultry because they did not use consecutive incubation at different pH values, temperatures, and enzymatic activity conditions similar to the different GI compartments emulated in this research. In this regard, Shar et al. [8] reported the application of banana peel as a biosorbent for in vitro removal of B-aflatoxins (0.25 µg). The authors stated that the adsorption was significantly increased above pH 6 (48.4%), suggesting that the interaction of the protonated banana peel was less favorable than the deprotonated adsorbent. At a pH value of 7.0, the adsorption of AFB_1_ and AFB_2_ on the surface of the banana peel was 65.9 and 57.6%, respectively. In another study, Avantaggiato et al. [40] reported that grape pomace (peel and pulp) was able to adsorb AFB_1_ to the same extent (up to 84.6%) in the pH range of 3–9. Moreover, employing a standard biosorption methodology, our research group reported AFB_1_ uptakes up to 86% when using *Pyracantha koidzumii* biomasses. In the in vitro experiment, the biosorbents were used at 0.5% w/v in samples containing 100 ng/mL of B-aflatoxin standards [7]. Recently, Solís-Cruz et al. [41] evaluated the AFB_1_-adsorption capacity of chitosan and three cellulosic polymers using an in vitro digestive model. The researchers concluded that the best adsorbent materials were sodium carboxymethylcellulose (44.6%) and hydroxypropyl methylcellulose (43.1%). Avantaggiato et al. [40] also evaluated aflatoxin adsorption onto a carbon/aluminosilicate-based product (Standard Q/FIS) using a model simulating the in vivo conditions of the GI tract of swine. Authors reported that when the Standard Q/FIS was added to the diet (up to 2%, *w*/*w*), mycotoxin absorption was significantly reduced in a dose-dependent response—up to 88% for AFB_1_, 44% for zearalenone, and 29% for fumonisins and ochratoxins. These results are consistent with our findings.

To the best of our knowledge, this is the first report on the removal of AFB_1_ by the three different biosorbents using a multicompartmental model simulating the dynamic conditions in the GI tract of poultry. Due to the presence of higher quantities of functional groups, the tested biosorbents probably bind other mycotoxins and could be used in reducing both individual and combined negative effects of mycotoxins. However, further in vivo studies need to be conducted to prove their effectiveness in reducing the toxic effects of mycotoxins without compromising micronutrient bioavailability. Research in this direction is in progress.

## 3. Materials and Methods

### 3.1. Chemical and Reagents

Chemicals and reagents were of analytical grade. Aflatoxin B_1_ (AFB_1_; ≥98% purity; CAS number 1162-65-8), HPLC grade methanol (CH_3_OH; ≥99.9% purity; CAS number 67-56-1), sodium hydroxide (NaOH; ≥97% purity; CAS number 1310-73-2), dimethyl sulfoxide (DMSO; ≥99.9% purity; CAS number 67-68-5), hydrochloric acid (HCl; ~37% purity; CAS number 7647-01-0), and sodium bicarbonate (NaHCO_3_; CAS number 144-55-8) were obtained from Merck KGaA (Darmstadt, Germany). Pepsin 1:10,000 and pancreatin 8× were obtained from Bio Basic (Markham, ON, Canada). Bromine solution (0.03%) was procured from Vicam (Milford, MA, USA).

### 3.2. Safety Precautions

Techniques used for handling and detoxifying AFB_1_-contaminated materials were implemented from the recommendations published by Castegnaro et al. [42].

### 3.3. Plant Material and Preparation of Adsorbents

Formosa firethorn [*Pyracantha koidzumii* (Hayata) Rehder] and *Aloe vera* (*A. barbadensis* Miller), cultivated in the Botanic Garden of the National Autonomous University of Mexico—Superior Studies Faculty at Cuautitlan were collected during March–April 2018. The method for *P. koidzumii* biosorbent production has been previously described in detail by Ramales-Valderrama et al. [7]. Briefly, the most recently matured leaves were washed with distilled water and dried (Binder model RE-115, Tuttlingen, Germany) at 40 °C for 24 h, grinded (Glen Mills Inc., Clifton, NJ, USA) and sieved (60 mesh) to provide enough material with a particle size of <250 µm. For *A. vera*, matured leaves were cut from the middle, and the gel was separated by scratching. The *Aloe* gel was pasteurized at 65 °C for 15 min and further concentrated utilizing low-temperature evaporation. Finally, samples were milled to obtain a white to light-cream fine powder. Banana (*Musa sapientum* L. *var. paradisiaca*) fruits were sourced from a local fruit market. The unripe bananas were washed with distilled water, and the peel was separated manually. Peels were cut into small pieces (1 cm^2^), dried, and milled using the same process conditions as indicated for *P. koidzumii* adsorbent preparation. The three biosorbents were stored at −20 °C until assayed. A noncommercial zeolitic mineral from the state of Guerrero, Mexico was used as a reference. This material is referred to in this article as zeolite.

### 3.4. Biosorbent Characterizations

#### 3.4.1. Zeta Potential (ζ)

Zeta potential measurements were conducted on a Zetasizer Nano Series ZSP system (Malvern Instruments, Worcestershire, UK) following the recommendations of Ramales-Valderrama et al. [7]. In addition, the zeta potential was determined at the pH value of each compartment simulated in the in vitro digestion model.

#### 3.4.2. Point of Zero Charge (pH_pzc_)

The pH_pzc_ was determined following the recommendations of Shar et al. [8] with minimal modifications. pH measurements were performed using a combination glass electrode (Conductronic PC-45, Puebla, Mexico). Briefly, 25 mL of distilled water was adjusted to the required pH value (2, 4, 6, 8, 10, and 12) by the addition of 0.1 M HCl or 0.1 M NaOH. The solutions at each pH were added into flasks containing preweighed biosorbents (0.425 g) and stirred at 250 rpm for 195 min. The final pH (pH_f_) was evaluated, and the difference (ΔpH) was calculated. ΔpH was plotted against the initial pH (pH_i_), and the point where the line crosses the *x*-axis gave the pH_pzc_ of the biosorbent.

#### 3.4.3. Energy-Dispersive X-ray Spectroscopy (EDS)

The multielemental analysis was carried out using an environmental scanning electron microscope accessorized with energy-dispersive X-ray spectroscopy (Phillips XL30/40 EDS-ESEM, Eindhoven, The Netherlands). Triplicates of each sample were evaluated using a high-performance XTrace microspot X-ray source, and the generated X-ray fluorescence spectrum was measured with the attached XFlash^®^ 6/10 silicon drift detector (Bruker Nano GmbH, Berlin, Germany).

#### 3.4.4. X-Ray Diffraction (XRD)

The biosorbents were studied by X-ray diffractometry according to the recommendations of Hernández-Meléndez et al. [43]. The XRD patterns were collected in the region of 2θ from 10° to 80° using a 0.02° step size.

#### 3.4.5. Fourier Transform Infrared Spectroscopy with Attenuated Total Reflection (FTIR-ATR)

Biosorbents were characterized using a Frontier SP8000 FTIR spectrophotometer (Perkin Elmer, Waltham, MA, USA) following the recommendations of Estrada-Urbina et al. [44]. All the spectra were taken in the range of 4000–400 cm^−1^.

#### 3.4.6. Diffuse Reflection UV-Vis

The diffuse reflectance of the biosorbents was performed using a Lambda 365 UV-Vis spectrophotometer (Perkin Elmer, Waltham, MA, USA) equipped with an integrating sphere to capture diffusely reflected light. A barium sulfate (BaSO_4_) reference was used to provide 100% reflectance. The optical absorption spectra were collected in the 300–700 nm range and subsequently converted to the Kubelka–Munk function [45].

### 3.5. Adsorption Experiments

#### 3.5.1. Aflatoxin Stock Solution

A primary standard solution of AFB_1_ (100 μg/mL) was prepared in DMSO; subsequently, the aflatoxin solution was diluted to 1 μg/mL using distilled water.

#### 3.5.2. Preparation of the AFB_1_-Contaminated Diet

A typical maize–soybean meal diet containing 19.5% protein and 13 MJ/kg metabolizable energy was prepared based on the National Research Council recommendations [46]. The compositional and chemical analysis of the diet is presented in Table S1. No antibiotic growth promoters or anticoccidial drugs were added to the diet. Samples were artificially contaminated by spiking 5 g of feed with 0.5 mL of the aflatoxin solution in order to reach an aflatoxin content of 100 μg/kg. The aflatoxin-contaminated samples were left overnight at 40 °C to allow residual solvent evaporation. Finally, 5 samples were randomly taken, and the AFB_1_ content was analyzed using the 991.31 AOAC method described below [47].

#### 3.5.3. In Vitro Digestive Model

A laboratory model to simulate the GI conditions of poultry was used to evaluate the efficacy of the biosorbents in reducing the AFB_1_ biodisponibility in the intestine. The setup of the GI model has been described in detail by Solis-Cruz et al. [41] and Hernandez-Patlán et al. [48]. All treatments were done in quintuplicate at 40 °C to simulate poultry body temperature.

#### 3.5.4. Aflatoxin Assay

Aflatoxins were extracted from samples using the Official AOAC method 991.31 [47]. Briefly, 1 mL of filtered supernatant was passed through the immunoaffinity column (Afla B, VICAM Science Technology, Watertown, MA, USA) followed by fluorometric detection (VICAM Source Scientific, Irvine, CA, USA). Methanolic extracts (200 µL) were further characterized using a fluorescence LS-55 spectrophotometer (Perkin Elmer, Waltham, MA, USA). Spectra were acquired in the 350–600 nm range using a 96-well plate reader accessory. The fluorescence emission spectra were collected at an excitation wavelength of 365 nm. The detection limit for AFB_1_ is estimated to be 0.5 ng/mL.

### 3.6. Experimental Design and Statistical Analysis

A completely randomized design with 5 replicates was used. Experimental data were subjected to one-way analysis of variance (ANOVA), and the means were compared by the Tukey test. A probability *p* < 0.05 was used to distinguish significant differences employing the Statistical Analysis System [49].

## Figures and Tables

**Table 1:** The elemental composition (%) of biosorbents and the inorganic mycotoxin binder (zeolite).

Element	Adsorbent			
	Banana	*Pyracantha*	*Aloe*	Zeolite
C	54.30 ± 0.20 ^a^	67.35 ± 0.52 ^b^	36.14 ± 0.79 ^c^	4.30 ± 0.8 ^d^
O	42.99 ± 0.13 ^a^	31.86 ± 0.48 ^b^	49.54 ± 0.70 ^c^	65.10 ± 0.6 ^d^
Na	0.03 ± 0.01 ^a^	ND	2.08 ± 0.06 ^b^	0.97 ± 0.03 ^c^
Mg	0.06 ± 0.01 ^a^	0.09 ± 0.01 ^a^	0.86 ± 0.02 ^b^	0.52 ± 0.04 ^c^
Al	0.05 ± 0.01 ^a^	ND	0.10 ± 0.06 ^a^	5.29 ± 0.04 ^b^
Si	0.08 ± 0.01 ^a^	0.03 ± 0.01 ^a^	0.19 ± 0.01 ^a^	20.86 ± 0.40 ^b^
P	0.10 ± 0.01 ^a^	0.07 ± 0.01 ^a^	1.09 ± 0.06 ^b^	ND
S	0.04 ± 0.01 ^a^	0.07 ± 0.01 ^a^	0.17 ± 0.01 ^b^	0.19 ±0.01 ^b^
Cl	0.38 ± 0.01 ^a^	ND	4.27 ± 0.29 ^b^	ND
K	1.89 ± 0.01 ^a^	0.31 ± 0.01 ^b^	4.10 ± 0.33 ^c^	0.75 ± 0.01 ^b^
Ca	0.05 ± 0.01 ^a^	0.21 ± 0.01 ^a^	1.47 ± 0.13 ^b^	1.45 ± 0.02 ^b^
Fe	ND	ND	ND	0.59 ± 0.04

Mean values ± standard error. Means with a different letter in the same row are statistically different (Tukey *p* < 0.05). ND = Not detected.

**Table 2:** Band assignments of the vibrational frequencies in the biosorbents.

Band	Wavenumber (cm^−1^)			Functional Group and Commonly Assigned Compound
	Banana	*Pyracantha*	*Aloe*	
A	-	-	3685 _(vw)_	O–H stretching (inner-layer Al–O…H)
B	-	-	3674 _(vw)_	O–H stretching (Mg^2+^–OH)
C	-	-	3660 _(vw)_	O–H stretching (Al^3+^ Mg^2+^–OH)
D	3271 _(br)_	3293 _(br)_	3240 _(br)_	N–H stretching vibrations (peptide and protein) O–H stretch
E	2922 _(m)_	2919 _(m)_	2976 _(m)_	CH_2_ antisymmetric stretching (lipids)
F	2854 _(w)_	2860 _(m)_	2902 _(m)_	C–CH_3_ symmetric stretching (lipids)
G	1738 _(w)_	1736 _(m)_	1721 _(m)_	C=O stretching (phospholipid esters)
H	-	1686 _(w)_	-	O=C–N–H (80% C=O stretching, 20% C–N stretching) (amide I, peptide, protein)
I	1598 _(br)_	1607 _(br)_	1587 _(s)_	Aromatic C=C stretch (lignin)
J	-	1516 _(w)_	-	NH_3_^+^ deformation (amino acid)
K	1436 _(w)_	1441 _(m)_	-	CH_3_ antisymmetric bending (lipid, protein) CH_2_ antisymmetric bending (lipid, protein)
L	-	-	1392 _(s)_	CH_3_ symmetric bending, C=O stretching (lipid, protein)
M	1370 _(w)_	1371 _(m)_	-	C–CH_3_ wagging, twisting and rocking symmetric (phospholipid, fatty acid, triglyceride)
N	1308 _(m)_	-	-	N–H rocking, C–N stretching, C=O rocking, C–C stretching and CH_3_ stretching (amide III, peptide, protein)
O	1245 _(w)_	1243 _(m)_	1254 _(m)_	PO_2_^−^ antisymmetric stretching (DNA, RNA, phospholipid, phosphorylated protein)
P	1149 _(m)_	1159 _(m)_	-	C–O stretching, C–OH wagging, twisting and rocking (carbohydrates)
Q	1073 _(s)_	1091 _(s)_	1076 _(vs)_	(PO_2_^−^) symmetric stretching (DNA, RNA, phospholipid, phosphorylated protein) Si–O stretch
R	1000 _(vs)_	1042 _(vs)_	1050 _(vs)_	C–O stretching (carbohydrate) C–O–P stretching (phosphate ester)
S	861 _(m)_	830 _(w)_	894 _(m)_	CH out-of-plane deformation NH_2_ wag (primary amines) Si–C stretch
T	767 _(m)_	767 _(w)_	772 _(w)_	CH out-of-plane deformation
U	-	610 _(w)_	639 _(w)_	C–CO–C bend
V	572 _(m)_	562 _(w)_	-	In-plane and out-of-plane ring deformations
W	522 _(m)_	-	527 _(s)_	In-plane and out-of-plane ring deformations C–Cl stretch

s = strong; m = medium; w = weak; v = very; br = broad.

**Table 3:** Band assignments of the vibrational frequencies in the zeolite.

Band	Wavenumber (cm^−1^)	Functional Group and Commonly Assigned Compound
A	3684 _(vw)_	O–H stretching (inner-layer Al–O…H)
B	3675 _(vw)_	O–H stretching (Mg^2+^–OH)
C	3658 _(vw)_	O–H stretching (Al^3+^ Mg^2+^–OH)
D	3624 _(vw)_	O–H stretching (Al^3+^–OH)
E	3568 _(vw)_	O–H stretching (Fe^3+^–OH)
F	3410 _(w)_	O–H stretching (H–O–H, surface oxygen)
G	2982 _(m)_	–CH_3_ and –CH_2_ in aliphatic hydrocarbons (presence of the organic component)
H	2899 _(m)_	–CH_3_ and –CH_2_ in aliphatic hydrocarbons (presence of the organic component)
I	1631 _(m)_	H–O–H bending (liquid water)
J	1453 _(m)_	CH_3_ in aliphatic compounds. Antisymmetric CH_3_ deformation CH_2_ in aliphatic compounds. CH_2_ bending (scissors) vibration
K	1013 _(vs)_	Si–O–Si antisymmetric stretch
L	875 _(w)_	O–H bending (Fe^3+^–Al^3+^–OH)
M	794 _(m)_	Si–O–Si (SiO_4_ tetrahedral rings and quartz) stretching symmetric
N	597 _(m)_	SiO_4_ and AlO_4_ tetrahedral
O	522 _(m)_	Si–O bending vibration (polymerization of the SiO_4_–4 units)
P	465 _(s)_	Si–O bending mode (–SiO_4_–)

s = strong; m = medium; w = weak; v = very; br = broad.

**Table 4:** The adsorption capacity of biosorbents and the inorganic mycotoxin binder (zeolite) against aflatoxin B_1_ (AFB_1_) using an in vitro gastrointestinal model.

Adsorbent	AFB_1_ Content (ng/mL)	Adsorption (%)
Blank	27.0 ± 0.58 ^a^	0
Banana	19.5 ± 1.44 ^b^	27.78
*Pyracantha*	14.5 ± 0.29 ^c^	46.30
*Aloe*	8.5 ± 0.87 ^d^	68.52
Zeolite	8.0 + 0.95 ^d^	70.19

Mean values ± standard error. Means with different letter in the same row are statistically different (Tukey *p* < 0.05).

## Supplementary Materials

The following are available online at http://www.mdpi.com/2072-6651/10/11/484/s1, Table S1: Ingredient composition of the experimental diet.
