# Supplementary material for: Assessing the Aflatoxin B1 Adsorption Capacity between Biosorbents Using an In Vitro Multicompartmental Model Simulating the Dynamic Conditions in the Gastrointestinal Tract of Poultry
Source: Toxins (Basel). 2018 Nov 21;10(11):484. doi: 10.3390/toxins10110484 (PMC6265716; doi:10.3390/toxins10110484)
Supplement: Supplementary file 1 [file toxins-10-00484-s001.pdf]

Supplementary Materials: Assessing the Aflatoxin B_1 Adsorption Capacity between Biosorbents Using an In Vitro Multicompartmental Model Simulating the Dynamic Conditions in the Gastrointestinal Tract of Poultry

Anai Zavala-Franco, Daniel Hernández-Patlán, Bruno Solís-Cruz, Raquel López-Arellano, Guillermo Tellez-Isaias, Alma Vázquez-Durán and Abraham Méndez-Albores

Table S1. Ingredients of the experimental diet.

**Ingredient**	**%**
Maize	54.64
Soybean meal	36.94
Vegetable oil	3.32
Dicalcium phosphate	1.58
Calcium carbonate	1.44
Salt	0.35
DL-Methionine	0.25
Choline chloride 60%	0.20
L-Lysine HCl	0.10
Vitamin premix [1]	0.30
Mineral premix [2]	0.30
Antioxidant [3]	0.15

[1] Vitamin premix supplied the following per kg: vitamin A, 20,000,000 IU; vitamin D3, 6,000,000 IU; vitamin E, 75,000 IU; vitamin K3, 9 mg; thiamine, 3 mg; riboflavin, 8 mg, pantothenic acid, 18 mg; niacin, 60 mg; pyridoxine, 5 mg; folic acid, 2 mg; biotin, 0.2 mg; cyanocobalamin, 16 mg; and ascorbic acid, 200 mg. [2] Mineral premix supplied the following per kg; manganese, 120 mg; zinc, 100 mg; iron, 120 mg; copper, 10-15 mg; iodine, 0.7 mg; selenium, 0.4 mg; and cobalt, 0.2 mg. [3] Ethoxyquin.
